# Single Subcutaneous Injection of Lysophosphatidyl-Choline Evokes ASIC3-Dependent Increases of Spinal Dorsal Horn Neuron Activity

**DOI:** 10.3389/fnmol.2022.880651

**Published:** 2022-06-14

**Authors:** Ludivine Pidoux, Kevin Delanoe, Julie Barbier, Fabien Marchand, Eric Lingueglia, Emmanuel Deval

**Affiliations:** ^1^Université Côte d’Azur, CNRS, IPMC, LabEx ICST, FHU InovPain, Valbonne, France; ^2^Université Clermont Auvergne, Inserm U1107 Neuro-Dol, Pharmacologie Fondamentale et Clinique de la Douleur, Clermont-Ferrand, France

**Keywords:** pain, lysophosphatidyl-choline, acid-sensing ion channel 3, spinal cord neurons, sodium channel, TRPV1

## Abstract

Lysophosphatidyl-choline (LPC), a member of the phospholipid family, is an emerging player in pain. It is known to modulate different pain-related ion channels, including Acid-Sensing Ion Channel 3 (ASIC3), a cationic channel mainly expressed in peripheral sensory neurons. LPC potentiates ASIC3 current evoked by mild acidifications, but can also activate the channel at physiological pH. Very recently, LPC has been associated to chronic pain in patients suffering from fibromyalgia or osteoarthritis. Accordingly, repetitive injections of LPC within mouse muscle or joint generate both persistent pain-like and anxiety-like behaviors in an ASIC3-dependent manner. LPC has also been reported to generate acute pain behaviors when injected intraplantarly in rodents. Here, we explore the mechanism of action of a single cutaneous injection of LPC by studying its effects on spinal dorsal horn neurons. We combine pharmacological, molecular and functional approaches including *in vitro* patch clamp recordings and *in vivo* recordings of spinal neuronal activity. We show that a single cutaneous injection of LPC exclusively affects the nociceptive pathway, inducing an ASIC3-dependent sensitization of nociceptive fibers that leads to hyperexcitabilities of both high threshold (HT) and wide dynamic range (WDR) spinal neurons. ASIC3 is involved in LPC-induced increase of WDR neuron’s windup as well as in WDR and HT neuron’s mechanical hypersensitivity, and it participates, together with TRPV1, to HT neuron’s thermal hypersensitivity. The nociceptive input induced by a single LPC cutaneous rather induces short-term sensitization, contrary to previously described injections in muscle and joint. If the effects of peripheral LPC on nociceptive pathways appear to mainly depend on peripheral ASIC3 channels, their consequences on pain may also depend on the tissue injected. Our findings contribute to a better understanding of the nociceptive signaling pathway activated by peripheral LPC *via* ASIC3 channels, which is an important step regarding the ASIC3-dependent roles of this phospholipid in acute and chronic pain conditions.

## Introduction

Lysophosphatidyl-choline (LPC) is an emerging lipid involved in pain (Gentry et al., 2010; Marra et al., 2016; Hung et al., 2020; Rimola et al., 2020; Sadler et al., 2021; Jacquot et al., 2022). It is an endogenous lysophospholipid that can be produced following plasma membrane hydrolysis due to PLA2 enzymes (Murakami et al., 2020) or oxydative stress (Karabina and Ninio, 2006; Choi et al., 2011), but it also serves as an intermediate for the synthesis of phosphatidyl-choline (PC) lipids (D’Arrigo and Servi, 2010). We initially identified LPC in the synovial fluids of patients suffering from painful joint diseases, as a positive modulator of the pain-related Acid-Sensing Ion Channel 3 (ASIC3) (Marra et al., 2016). More recently, we demonstrated that the synovial fluid levels of LPC16:0 species was correlated with pain outcomes in patients with osteoarthritis (Jacquot et al., 2022), and a correlation between the serum levels of LPC16:0 and pain symptoms has also been found in fibromyalgia patients (Hung et al., 2020). Interestingly, injecting LPC16:0 in either muscles (Hung et al., 2020) or joints (Jacquot et al., 2022) generates ASIC3-dependent persistent pain-like states in mice, indicating that this LPC species is a potential triggering factor of chronic pain associated to human musculoskeletal diseases, at least in osteoarthritis (Jacquot et al., 2022) and fibromyalgia (Hung et al., 2020). LPC has also been shown to generate acute pain when injected cutaneously/intraplantarly in rodents (Gentry et al., 2010; Marra et al., 2016; Rimola et al., 2020), including ASIC3-dependent acute pain-like behaviors (Marra et al., 2016).

Acid-sensing ion channel 3 belongs to the Acid-Sensing Ion Channels’ family, which are depolarizing cation channels known for their ability to sense extracellular protons (Waldmann et al., 1997a). Several ASIC subunits have been identified in mammals, including ASIC1, ASIC2, ASIC3, and ASIC4, with several variants [for reviews, see Deval and Lingueglia (2015) and Lee and Chen (2018)]. A functional ASIC channel results from the trimeric assembly of these subunits (Jasti et al., 2007), at least for ASIC1, ASIC2, and ASIC3, leading to homomeric and/or heteromeric channels with different biophysical properties and regulations (Hesselager et al., 2004). ASICs are widely distributed in the nervous system and all along the pain pathway. Most ASIC subunits are expressed in sensory neurons, where ASIC3 and ASIC1b subunits have been shown to be important players in several pain models (Sluka et al., 2003; Deval et al., 2008, 2011; Diochot et al., 2012, 2016; Verkest et al., 2018; Chang et al., 2019). If ASICs are extracellular pH sensors, their activity and/or expression are nevertheless highly regulated by various endogenous factors associated with ischemia, inflammation, and pain (Immke and McCleskey, 2001; Mamet et al., 2002; Deval et al., 2004, 2008; Sherwood and Askwith, 2009; Li et al., 2010). This is particularly true for ASIC3 channels (Waldmann et al., 1997b), which seems to behave as “coincidence detectors” of several pain-related mediators, including mild extracellular acidification, hypertonicity, ATP and/or lipids (Deval et al., 2008; Birdsong et al., 2010; Li et al., 2010). LPC, alone or in combination with arachidonic acid (AA), induced a sustained ASIC3 current at physiological pH 7.4, in addition to the potentiation of its current evoked by mild acidifications (Marra et al., 2016; Jacquot et al., 2022).

Here, we investigate how a hindpaw local cutaneous injection of LPC affects the nociceptive pathway and the activity of spinal dorsal horn neurons, as well as the contribution of ASIC3 to this process. We combine *in vivo* and *in vitro* approaches to (i) explore the mechanism of action associated with LPC effect, (ii) determine the role of peripheral ASIC3 to the generation of the pain message, and (iii) study how this message is integrated at the spinal cord level. We show that LPC, which activates and potentiates ASIC3 *in vitro*, positively modulates both spontaneous and evoked activities of particular subsets of spinal dorsal horn neurons. Cutaneous hindpaw injection of LPC in rats or mice enhances the firing of high threshold (HT) and wide-dynamic range (WDR) neurons, leaving low threshold (LT) neurons unaffected. Hindpaw LPC injection is associated to short-term sensitization of nociceptive fibers, which is significantly reduced by the local pharmacological inhibition of ASIC3, and almost abolished in ASIC3 knockout mice. This work shows how a single local cutaneous administration of LPC induces peripheral sensitization of ASIC3-expressing nociceptive fibers that drive hyperexcitability of neurons within the dorsal spinal cord.

## Materials and Methods

### Cell Culture and Transfections

HEK293 cell line was grown in DMEM medium supplemented with 10% of heat-inactivated fetal bovine serum (BioWest) and 1% of antibiotics (penicillin + streptomycin, BioWhittaker). One day after plating, cells were transfected with either pIRES2-rASIC1a-EGFP (rat ASIC1a), pIRES2-rASIC1b-EGFP (rat ASIC1b), or pIRES2-rASIC3-EGFP (rat ASIC3) vectors using the JetPEI reagent according to supplier’s protocol (Polyplus transfection SA, Illkirch, France). Fluorescent cells were used for patch clamp recordings 2–4 days after transfection.

### Patch Clamp Experiments

Whole cell configuration of the patch clamp technique was used to record membrane currents at a holding potential of −80 mV (voltage clamp mode). Recordings were made at room temperature using an axopatch 200B amplifier (Axon Instruments) with a 2 kHz low-pass filter. Data were digitized by a Digidata 1550 A-D/D-A converter (Axon Instruments), sampled at 20 kHz and recorded on a hard disk using pClamp software (version 11; Axon Instruments). The patch pipettes (2–6 MΩ) were filled with an intracellular solution containing (in mM): 135 KCl, 2 MgCl_2_, 5 EGTA, and 10 HEPES (pH 7.25 with KOH). The extracellular solution bathing the cells contained (in mM) the following: 145 NaCl, 5 KCl, 2 MgCl_2_, 2 CaCl_2_, 10 HEPES (pH 7.4 with *N*-methyl-D-glucamine). ASIC currents were induced by shifting one out of eight outlets of a homemade microperfusion system driven by solenoid valves, from a holding control solution (i.e., pH 7.4) to an acidic test solution (pH 7.0 or pH 6.6). Cells were considered as positively transfected when they exhibited a visible GFP fluorescence and a transient pH 6.6-evoked current of at least 300 pA (IpH 6.6 ≥ 300 pA). Non-transfected (NT) cells were used as controls and they were selected in Petri dishes having undergone the transfection protocol described above, but with no visible GFP fluorescence and no significant pH 6.6-evoked current.

### Animals

Experiments were performed on adult male Wistar Han rats (Charles River, age > 6 weeks), adult male C57Bl6J wild type mice (WT, Janvier Lab, age > 7 weeks), and ASIC3 knockout mice (ASIC3 KO, internal animal husbandry, age > 7 weeks). The protocol was approved by the local ethical committee and the French government (agreement n° 02595.02). Animals were kept with a 12 h light/dark cycle with access to food and water *ad libitum*, and were acclimated to housing and husbandry conditions for at least a week before experiments.

### Surgery

Anesthesia was induced with a mix of air and isoflurane 4% (Anesteo, Lunel, France). Animals were then placed in a stereotaxic frame (M2E, Montreuil, France) and kept under anesthesia using a mask diffusing a mix of oxygen and isoflurane 2%. The head and vertebral column of the animal were stabilized by ear bars and vertebral clamps, respectively, while a limited laminectomy was performed between vertebrae T13 and L2. Dura was carefully removed, and the spinal cord was immerged with artificial cerebrospinal fluid (ACSF containing 119 mM NaCl, 2.5 mM KCl, 1.25 mM NaH_2_PO_4_, 1.3 mM MgSO_4_, 2.5 mM CaCl_2_, 26 mM NaHCO_3_, 11 mM glucose, and 10 mM HEPES, pH adjusted to 7.4 with NaOH) before starting electrophysiological recordings.

### *In vivo* Electrophysiological Recordings of Spinal Cord Neurons

Single-unit extracellular recordings of spinal dorsal horn neurons were made with tungsten paralyn-coated electrodes (0.5 MΩ, WPI, Hertfordshire, Europe) and using Spike2 acquisition system (Cambridge Electronic Design, Cambridge, United Kingdom). The tip of a recording electrode was initially placed on the dorsal surface of the spinal cord using a micromanipulator (M2E, Montreuil, France) and this initial position determined the zero on the micromanipulator’s micrometer. The electrode was then progressively moved down into the dorsal horn until the receptive field of a spinal neuron was localized on the ipsilateral plantar hindpaw using mechanical stimulations. Neuronal signals were bandpass filtered (0.3–30 kHz) and amplified using a DAM80 amplifier (WPI, Hertfordshire, Europe), digitized with a 1401 data acquisition system (Cambridge Electronic Design, Cambridge, United Kingdom), sampled at 20 kHz and finally stored on a computer.

**FIGURE 1:**
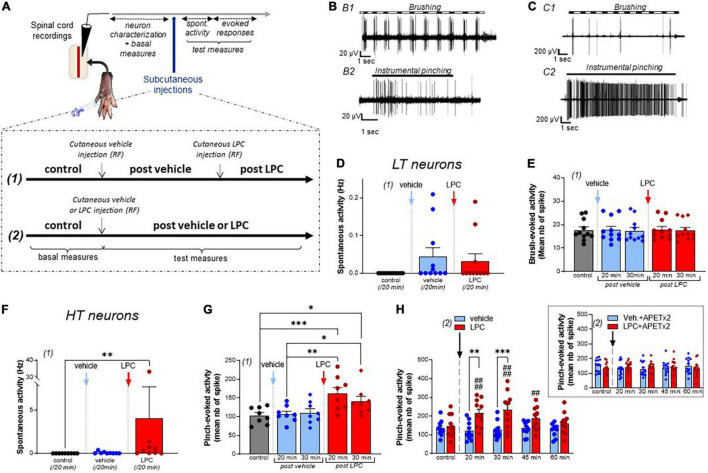
Effect of LPC cutaneous injection on low threshold (LT) and high threshold (HT) spinal neuron activity. **(A)** Protocol used for spinal dorsal horn neuron recordings. After neuron characterization in the spinal cord, vehicle, and/or LPC were subcutaneously injected in the hindpaw using two protocols described in the inset. **(B)** Typical discharge of a rat low threshold (LT) neuron responding to non-noxious brushing **(B1)**, but not to noxious pinching (**B2**, instrumental pinching, 300 g). **(C)** Typical discharge of a rat high threshold (HT) neuron that did not respond to brush **(C1)** but emitted a sustained discharge in response to pinch (**C2**, instrumental pinching, 300 g). **(D)** Global spontaneous activity of LT neurons assessed with protocol (1) over 20 min-periods following vehicle (blue bar and points) and LPC16:0 (red bar and points) subcutaneous injection within the neuron’s receptive fields (*n* = 11 neurons from 7 rats, Friedman test with *p* = 0.0988). **(E)** Brushing-evoked responses of LT neurons before (control) and after vehicle/LPC16:0 subcutaneous injection in their receptive fields [*n* = 11 neurons from 7 rats, no significant differences with *p* = 0.9822, Friedman test; protocol (1) used]. **(F)** Global spontaneous discharge of HT neurons after vehicle (blue bar and points) and LPC16:0 (red bar and points) injections [*n* = 9 neurons from 7 rats, Friedman test with *p* < 0.0001 followed by a Dunn’s multiple comparison test: **p = 0.0012; protocol (1) used]. **(G)** Pinch-evoked responses of HT neurons (instrumental pinching, 300 g) before and after vehicle/LPC16:0 subcutaneous injection in their receptive fields [*n* = 8 neurons from 6 rats, Friedman test with *p* < 0.0001 followed by a Dunn’s multiple comparison test: *p < 0.05, **p < 0.01, and ***p < 0.001; protocol (1) used]. **(H)** Duration of the LPC effect on pinch-evoked activity of HT neurons [*n* = 10 and 11 neurons for vehicle and LPC, respectively; two-way ANOVA test with *p* = 0.0156 and *p* < 0.0001 for treatment and time after injection, respectively; **p < 0.01 and ***p < 0.001, Sidak’s multiple comparison *post-hoc* test; ##*p* < 0.01 and ####*p* < 0.0001 compared to control before LPC injection, Dunnet’s multiple comparison *post-hoc* test; protocol (2) used]. The potentiation by cutaneous LPC was still significant 45 min after injection. Inset: The effect of LPC was abolished when APETx2 (0.2 nmol) was co-injected together with the lipid. Note that APETx2 had no effect by itself (vehicle + APETx2) on pinch-evoked activity of HT neurons [*n* = 13 and 15 for vehicle + APETx2 and LPC + APETx2, respectively; two-way ANOVA test with *p* = 0.8062 and *p* = 0.7850 for treatment and time after injection, respectively; protocol (2) used].

**FIGURE 2:**
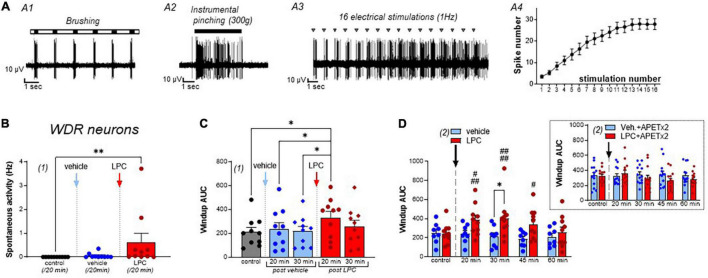
Effect of LPC cutaneous injection on spinal wide dynamic range (WDR) neuron activity. **(A)** Typical discharge of a rat wide dynamic range (WDR) neuron, which responded to both brushing **(A1)** and pinching (**A2**, instrumental pinching, 300 g). WDR neuron also exhibited windup **(A3)** following repetitive electrical stimulations of its receptive field (gray triangle). Typically, windup is characterized by a progressive increase in the number of C-fiber evoked spikes as the number of stimulations increase (**A4**, data from 22 neurons). Maximal windup was reached between the 13th and 16th stimulation, from 3.48 ± 0.72 spikes at the first stimulation to 27.86 ± 2.5 spikes at the 16th stimulation. **(B)** Global spontaneous discharge of WDR neurons after vehicle (blue bar and points) and LPC16:0 (red bar and points) subcutaneous injections in their receptive fields (*n* = 10 neurons from 8 rats, Friedman test with *p* = 0.0008 followed by a Dunn’s multiple comparison test: **p = 0.0036). **(C)** WDR neuronal windup before and after vehicle (blue bars and points) and LPC16:0 (red bars and points) subcutaneous injections in their receptive fields. C-fiber-induced windup is represented as the area under curve (AUC) determined from classical windup curves (see Section “Materials and Methods,” *n* = 10 neurons from 8 rats, Friedman test with *p* = 0.0111 followed by a Dunn’s multiple comparison test: *p < 0.05). **(D)** The potentiating effect of LPC on WDR neuronal windup was significant for 45 min (*n* = 10 neurons from 8 rats and *n* = 8 neurons from 7 rats for LPC and vehicle, respectively, two-way ANOVA test with *p* = 0.0835 and *p* = 0.0047 for treatment and time after injection, respectively; *p < 0.05, Sidak’s multiple comparison *post-hoc* test; #*p* < 0.05, ###*p* < 0.001, and ####*p* < 0.0001 compared to control before LPC injection, Dunnet’s multiple comparison *post-hoc* test), and was abolished when APETx2 (0.2 nmol) was co-injected together with LPC. Note that APETx2 had no effect by itself (vehicle + APETx2) on WDR neuron windup (inset: *n* = 13 and 13 for vehicle + APETx2 and LPC + APETx2, respectively; two-way ANOVA test with *p* = 0.4594 and *p* = 0.3816 for treatment and time after injection, respectively).

Once a spinal neuron was isolated with its receptive field, non-noxious (brushing) and noxious (pinching) stimulations were used to characterize the neuronal type (Figures 1A–C, 2A). Classically, spinal neurons were differentiated depending on the peripheral input received (Almeida et al., 2004; Xu and Brennan, 2009; Xu et al., 2013): (i) LT neurons (Figure 1B), receiving input from non-nociceptive fibers and essentially responding to non-noxious brushing (Figure 1B1), with only modest and non-dynamic responses to noxious pinching (Figure 1B2), (ii) HT neurons (Figure 1C), receiving input from nociceptive fibers and dynamically responding to noxious pinching by a high frequency activity lasting the whole time of the stimulation (Figures 1C1,C2), and (iii) WDR neurons (Figure 2A), responding to both noxious and non-noxious stimulations (Aβ, Aδ, and C inputs, Figures 2A1,A2), and known to exhibit a facilitatory process (Latremoliere and Woolf, 2009) called windup (Figures 2A3,A4).

### Stimulation Protocols of Dorsal Horn Neuron Receptive Fields

Receptive fields of dorsal horn neurons were stimulated every 10 min by applying 10 consecutive non-noxious brushings, using a soft paint brush, and/or 5 consecutive noxious pinches, using either calibrated forceps (300 g stimulations, Bioseb, Vitrolles, France) or classical forceps (Moria MC40/B, Fine Science Tools, Heidelberg, Germany) for rats and mice, respectively. In addition to these mechanical stimuli, receptive field of WDR neurons also received repetitive electrical stimulations (protocol of 16 supraliminar 4 ms pulses, Dagan S900 stimulator) to induce windup. Intensity of currents injected for windup was determined as the intensity required to evoke less than 10 action potentials (APs) at the first stimulation, corresponding to 1.2–3 times the AP thresholds.

The evoked responses of dorsal horn neurons to non-noxious and noxious mechanical stimulations were also assessed using von Frey filaments. Different filaments were used to determine the mechanical sensitivity of HT and WDR neurons in rats (1 g; 8 g; 26 g; 60 g; 180 g; 300 g) and mice (0.40 g, 1 g, 2 g, 4 g, 6 g, 8 g, 10 g, 15 g). Each filament was applied three times during 3 s.

Finally, the response of dorsal horn neurons to thermal stimulation was assessed by applying heat ramps onto animal hindpaws. Heat ramps were applied by running a trickle of warm water on the neuron’s receptive field using a temperature controller (CL-100, Warner Instruments, Holliston, MA, United States). A temperature probe was placed on the center of the receptive field and temperature was monitored for the entire duration of the experiment. Temperature was initially set at 30°C and heat ramps were delivered for 47 s up to 47°C, every 10 min, before and after injection of LPC or vehicle.

### Peripheral Injection of Lipids and Drugs

Subcutaneous injection in the receptive field of dorsal horn neurons (20 μl and 10 μl for rats and mice, respectively) was made using a 28-gauge needle connected to a 50 μl Hamilton syringe. LPC16:0 and LPC18:1 were purchased from Anvanti (Coger, France), prepared as stock solutions in ethanol, and injected either alone (4.8 nmoles and 9.6 nmoles diluted in NaCl 0.9% for rats and mice, respectively) or in combination with pharmacological inhibitors: APETx2 (0.2 nmoles; purchased from Smartox Biotechnology, France, and prepared as stock solution in NaCl 0.9%) or capsazepine (0.2 nmoles, purchased from Smartox Biotechnology, France, and prepared as stock solution in DMSO). Ethanol or DMSO, diluted in NaCl 0.9%, were used as vehicle control solutions (ranging from 0.48 to 5%).

### Spike Sorting and Analysis

Off-line analyses of *in vivo* electrophysiological recordings were made using Spike2 (Cambridge Electronic Design, Cambridge, United Kingdom) and Matlab (MathWorks, Natick, MA, United States) softwares. The spike sorting was first performed with Spike2, using principal component analysis of spike waveforms. The spikes and associated stimulation train were then exported to Matlab to perform further analysis. For each neuron, both spontaneous activities and evoked responses to noxious or non-noxious stimulations were quantified as the number of spikes emitted at rest and during the different stimulations, respectively. Matlab codes were used to calculate mean number of spikes. Spontaneous activities were calculated over 20 min periods starting immediately after peripheral injections. For non-noxious brushings, the mean number of spikes was calculated over 10 consecutive stimulations. For noxious pinching, the mean number of spikes was calculated over the 5 consecutive 5 s stimulations. For windup analysis of WDR neurons, each interval between repetitive electrical stimulations was divided into periods, so that the spikes evoked by Aδ and C-fibers can be distinguished (Supplementary Figure 4A). Indeed, spikes emitted within the 20–90 ms interval after the stimulation artifact were attributed to Aδ-fibers, whereas those emitted during the 90–1,000 ms interval were attributed to C-fibers (90–350 ms) and the after depolarization (AD) period (350–1,000 ms). Windup curves were established by counting the number of spikes emitted during C-fiber + AD periods for each of the 16 repetitive electrical stimulations. Windup was then expressed as the area under curve (AUC), which was calculated with the baseline set at the Y value corresponding to the first number of spikes for each windup protocol.

For experiments using von Frey filaments, the mean number of emitted spikes was calculated over three consecutive 3 s stimulations.

### c-Fos Immunohistochemistry on the Spinal Cord Following Intraplantar Vehicle or LPC16:0 Injection

Following intraplantar vehicle (*n* = 6) or LPC16:0 (*n* = 5) administration, mice were maintained under isoflurane (1.5%) anesthesia during 1 h. Mice were then terminally anesthetized using a mixture of ketamine/xylazine and quickly perfused transcardially with saline followed by 4% paraformaldehyde (PFA). The lumbar spinal cord was excised and post fixed in 4% PFA in phosphate buffer (0.1 M, pH 7.4) for 24 h at 4°C. After cryoprotection (PB-Sucrose 30%) for at least 48 h, samples were included in tissue freezing medium (O.C.T.). Twenty μm cryostat thick frozen sections of the lumbar spinal cord were processed, mounted on Superfrost slides, blocked with PBS, BSA 1% and, incubated with a rabbit primary antibody against c-Fos (1:1,000; 9F6#2250, Cell Signaling) in PBS + BSA 1% + Triton 0.2% overnight at room temperature following three washes in PBS. After washes in PBS, sections were incubated with the corresponding secondary antibody (1:1,000, AlexaFluor 488 Molecular Probes, United States). After PBS washes, sections were then cover-slipped with fluorescent mounting medium (Dako) and observed with Nikon Eclipse Ni-E microscope. Quantitative analyses were performed with NIS-Elements software and a minimum of 7 sections per animal (*n* = 5–6 per group) were quantified by a blinded investigator and an average of the number of c-Fos positive neurons of the ipsilateral and contralateral dorsal horn (layers I & II and IV & V) counts was taken.

### Statistical Analysis of Data

Graphs and statistical analysis were made using GraphPad Prism software (GraphPad Software, San Diego, CA, United States). Numerical values are given as mean ± SEM, unless otherwise stated. Statistical differences between sets of data were assessed using either parametric or non-parametric tests followed by *post-hoc* tests, when appropriate. In all cases, the significance level was set at *p* ≤ 0.05. Statistical test used and significant *p*-values are indicated in each figure legend.

## Results

### *In vivo* Cutaneous Injection of Lysophosphatidyl-Choline Affects Spinal High Threshold, but Not Low Threshold, Neurons

Lysophosphatidyl-choline has been associated to acute pain behaviors when injected cutaneously/intraplantarly in rodents (Gentry et al., 2010; Marra et al., 2016; Rimola et al., 2020). We thus performed *in vivo* recordings of spinal dorsal horn neuron activity to investigate how the acute pain message generated by subcutaneous injection of LPC, and more particularly LPC16:0 species (Marra et al., 2016; Jacquot et al., 2022), is integrated at the spinal level (see Section “Materials and Methods”). To determine whether LPC affects the firing of spinal dorsal horn neurons, both spontaneous activity and evoked neuronal responses to non-noxious and/or noxious stimuli were recorded in rats before and after vehicle or LPC injections (Figures 1A–C, 2A). Two different protocols were used for vehicle and LPC administration (Figure 1A): (1) consecutive administration of vehicle and LPC within the same animals, which allowed paired analyses, and (2) single administration of vehicle or LPC in different animals. With protocol (1), no significant effect of LPC on low threshold (LT) neurons was observed on either spontaneous activity (Figure 1D, 0.04 ± 0.02 Hz and 0.03 ± 0.02 Hz after vehicle and LPC16:0 injections, respectively), or non-noxious brush-evoked activity (Figure 1E). The spiking activity evoked by brushing remained unchanged 20 and 30 min after vehicle or LPC injection, compared to the evoked activity in control condition before any injection (Figure 1E, +0.6%, and −1.6% compared to control at 20 and 30 min, respectively, after vehicle injection, and +1.2% and −0.7% compared to control at 20 and 30 min, respectively, after LPC16:0 injection).

Lysophosphatidyl-choline was next tested on spinal high threshold (HT) neurons (Figures 1F–H). Both the spontaneous and pinch–evoked activities of HT neurons were significantly increased by LPC cutaneous injection (Figure 1F, Spontaneous activity: 0.07 ± 0.04 Hz for vehicle vs. 4.05 ± 3.67 Hz for LPC16:0; Figure 1G, Pinch-evoked activity: +3.8% and +7.4% compared to control at 20 and 30 min, respectively, after vehicle injection, and +57.1% and +36.5% compared to control at 20 and 30 min, respectively, after LPC16:0 injection). These results were confirmed using protocol (2) in which vehicle or LPC were administered in different animals. The HT neuron hyperexcitability induced by LPC lasted up to 45 min after its injection, demonstrating short-term sensitization to noxious mechanical stimuli (Figure 1H, −9.9, −7.5, +0.5, and −6.8% compared to control at 20, 30, 45, and 60 min, respectively, after vehicle injection, and +49.4, +61.9, +28.9, and +11.7% compared to control at 20, 30, 45, and 60 min, respectively, after LPC16:0 injection). A similar sensitization of HT neuron activity was also observed following cutaneous injection of LPC18:1 (Supplementary Figure 1A), another LPC species that has recently been involved in nociceptor activation and pain in rodents (Rimola et al., 2020). A maximal effect on HT neurons’ pinch-evoked activity was reached between ∼5 and ∼15 nmoles of LPC16:0 (Supplementary Figure 1B), with a decrease at 50 nmoles that could be related to desensitization or additional effects of LPC at high doses, for instance on the hyperpolarizing potassium channels TREK1 and TRAAK (Maingret et al., 2000).

Because LPC16:0 has been shown to activate/potentiate ASIC3 (Marra et al., 2016; Jacquot et al., 2022; Supplementary Figure 2), the ASIC3 blocker APETx2 (Diochot et al., 2004) was next co-injected with the lipid (Figure 1H, Inset). APETx2 prevented LPC-induced short-term sensitization of spinal HT neurons (−14.7, −14.7, −9.0, and −1.7% compared to control at 20, 30, 45, and 60 min, respectively, after vehicle + APETx2 injections, and +8.7, +5.5, +7.4, and −1.4% compared to control at 20, 30, 45, and 60 min, respectively, after LPC16:0 + APETx2 injections), supporting a role of ASIC3 channels in this effect. LPC has also been shown to activate some TRP channels, including TRPV1 and TRPM8 (Andersson et al., 2007; Gentry et al., 2010; Rimola et al., 2020). We thus tested the effect of capsazepine, which has been reported to block both channels (Weil et al., 2005; Messeguer et al., 2006). The hyperexcitability of HT neurons induced by peripheral injection of LPC was not significantly reduced by capsazepine (Supplementary Figure 3), suggesting that TRPV1 and TRPM8 channels were not involved in the increase of HT neuron spontaneous activity nor in their hypersensivity to pinch following cutaneous injection of LPC16:0.

### *In vivo* Cutaneous Injection of Lysophosphatidyl-Choline Affects Spinal Wide Dynamic Range Neurons

The spontaneous activity of spinal WDR neurons was significantly increased by LPC cutaneous injection, compared to vehicle (Figure 2B, 0.02 ± 0.01 Hz for vehicle vs. 0.66 ± 0.41 Hz for LPC16:0). Moreover, the C-fiber-evoked activity of WDR neurons was also enhanced by LPC, as illustrated by its effect on windup (Figures 2C,D and Supplementary Figures 4A,C), whereas WDR activity related to non-noxious brushing was unaffected (Supplementary Figures 4A,B, Aδ-evoked activity: 2.71 ± 0.26 spikes, 2.49 ± 0.29 spikes, and 3.10 ± 0.51 spikes for control, vehicle and LPC16:0 injection, respectively; Supplementary Figure 4D, Brush-evoked activity: 3.86 ± 0.24 spikes, 3.83 ± 0.36 spikes, and 4.47 ± 0.40 spikes for control, vehicle and LPC16:0 injection, respectively). Thus, LPC significantly increased windup compared to vehicle and control conditions (Figure 2C, +13.0% and +2.9% compared to control at 20 and 30 min, respectively, after vehicle injection, and +57.7% and +22.7% compared to control at 20 and 30 min, respectively, after LPC16:0 injection), with an effect that lasted at least 45 min (Figure 2D). Finally, as observed for the LPC potentiation of HT neuron evoked-activity, the potentiating effect on WDR neuron windup was abolished by APETx2, further supporting a role of ASIC3 channels (Figure 2D, Inset, −2.3, +4.5, +4.2, and +0.6% compared to control at 20, 30, 45, and 60 min, respectively, after vehicle + APETx2 injections, and +11.4, −10.6, −19.8, and −14.3% compared to control at 20, 30, 45, and 60 min, respectively, after LPC16:0 + APETx2 injections).

### Lysophosphatidyl-Choline-Induced Mechanical Hypersensitivity of Spinal High Threshold Neurons Is Dependent on Peripheral Acid-Sensing Ion Channel 3

**FIGURE 3:**
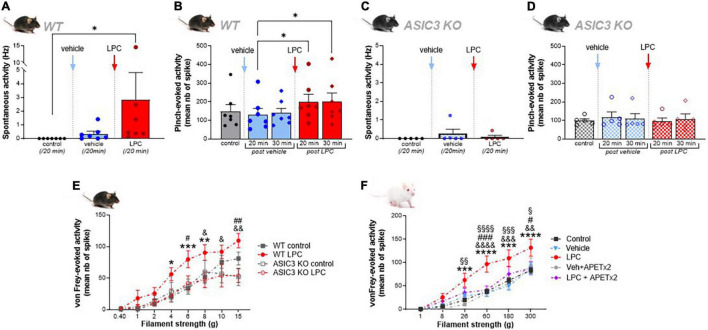
Effect of LPC cutaneous injection on spinal HT neuron on both spontaneous and mechanically evoked activity. **(A)** Global spontaneous discharge of HT neurons before and after vehicle (blue bar and points) and LPC (red bar and point) subcutaneous injections in wild-type mice receptive fields (*n* = 7 neurons from 6 WT mice, Friedman test with *p* < 0.0001 followed by a Dunn’s multiple comparison test: *p < 0.05 for control vs. LPC comparison). **(B)** Evoked responses of HT neurons to nociceptive stimulation (pinch) in WT mice after vehicle (blue bars and points) and LPC16:0 (red bars and points) subcutaneous injections. Effects were measured 20 min (circle point) and 30 min (diamond point) after respective injections (*n* = 7 neurons from 6 WT mice, Friedman test with *p* = 0.0025 followed by a Dunn’s multiple comparison test: *p < 0.05). **(C)** Global spontaneous discharge of HT neurons of ASIC3 KO mice before and after vehicle (blue bar and points) and LPC (red bar and points) subcutaneous injections (*n* = 5 neurons from 4 ASIC3 KO mice, no significant difference, *p* = 0.3333, Friedman test). **(D)** HT neuron responses of ASIC3 KO mice to nociceptive stimulation (pinch) before and after vehicle and LPC16:0 subcutaneous injections (*n* = 5 neurons from 4 ASIC3 KO mice, no significant difference, Friedman test with *p* = 0.3848). **(E)** Curves representing the mechanical sensitivity of mouse HT neurons following von Frey filament applications on their receptive fields before (black curves) and after LPC16:0 injection (red curves). Experiments were performed in both WT (full symbols, *n* = 15 neurons from 10 mice and 10 neurons from 6 mice for control and LPC, respectively) and ASIC3 KO mice (empty symbols, *n* = 5 neurons) (two way ANOVA with *p* = 0.0106 and *p* < 0.0001 for treatment and von Frey filaments effects, respectively, followed by Tukey’s multiple comparison test: *p < 0.05, **p < 0.01, and ***p < 0.001 for WT LPC vs. WT control; &*p* < 0.05 and &&*p* < 0.01 for WT LPC vs. ASIC3 KO LPC; #*p* < 0.05 and ##*p* < 0.01 for WT LPC vs. ASIC3 KO control). **(F)** Mechanical sensitivity of rat HT neurons to von Frey filaments before (control, *n* = 39 neurons) and after injection of vehicle (*n* = 10 neurons from 9 rats), vehicle + APETx2 (*n* = 9 neurons from 5 rats), LPC (*n* = 10 neurons from 9 rats), and LPC + APETx2 (*n* = 10 neurons from 6 rats; two way ANOVA with *p* = 0.0004 and *p* < 0.0001 for treatment and von Frey filaments effects, respectively, followed by Tukey’s multiple comparison test: ***p < 0.001 and ****p < 0.0001 for LPC vs. control; &&*p* < 0.01, &&&*p* < 0.001, and &&&&*p* < 0.0001 for LPC vs. vehicle; #*p* < 0.05 and ###*p* < 0.001 for LPC vs. LPC + APETx2; § *p* < 0.05, §§ *p* < 0.01, §§§ *p* < 0.001, and §§§§ *p* < 0.0001 for LPC vs. vehicle + APETx2).

To further explore the contribution of ASIC3 in the effect of cutaneous LPC, experiments were performed in WT and ASIC3 knockout mice. The spontaneous and evoked activities of HT neurons were both significantly potentiated following LPC cutaneous injection in WT mice (Figures 3A,B), whereas those of LT neurons remained unaffected (Supplementary Figures 5A,B), as observed in rats. HT neuron spontaneous activity was significantly higher following LPC injection compared to vehicle (Figure 3A, 0.23 ± 0.12 Hz for vehicle vs. 0.53 ± 0.17 Hz for LPC16:0). Moreover, response of HT neurons to noxious pinch in WT mice was also enhanced by LPC, with a 52.4% and 53.9% increase of evoked-activity 20 and 30 min, respectively, after LPC16:0 injection, compared to vehicle (Figure 3B). This potentiating effect of LPC lasted up to 45 min (Supplementary Figure 5E), similarly to what has been observed in rats (Figure 1H). Importantly, both effects on spontaneous and pinch-evoked activities of HT neurons were lost in ASIC3 knockout mice (ASIC3 KO, Figure 3C, 0.25 ± 0.23 Hz for vehicle vs. 0.09 ± 0.09 Hz for LPC16:0; Figure 3D, +19.2% and +10.2% compared to control at 20 and 30 min, respectively, after vehicle injection, −3.3% and +9.2% compared to control at 20 and 30 min, respectively, after LPC16:0 injection), further supporting the involvement of ASIC3 channels in LPC-induced hyperexcitability of spinal HT neurons.

The mechanical sensitivity of spinal HT neurons was next assessed using von Frey filaments in both mice (Figure 3E) and rats (Figure 3F). A set of filaments ranging from 0.4 to 15 g was applied successively (see Section “Materials and Methods”) in both WT and ASIC3 KO mice, before and after LPC cutaneous injection into HT neuron receptive fields (Figure 3E). Before LPC injection, HT neurons of both genotypes responded similarly to von Frey stimulations, with an increase of emitted spikes as a function of filament strength (Figure 3E, From 0.31 ± 0.21 to 81.04 ± 10.19 spikes for WT mice, and from 1.2 ± 1.12 to 51.87 ± 13.74 spikes for ASIC3 KO mice), showing no significant difference in their basal mechanical sensitivities. Following LPC injection, the mechanical sensitivity of WT HT neurons was significantly increased from filaments ≥ 4 g, an effect that was not observed in ASIC3 KO mice (Figure 3E, After LPC16:0 injection: from 1.63 ± 1.38 to 109.50 ± 11.48 spikes for WT mice, and from 2.26 ± 2.10 to 53.93 ± 10.97 spikes for ASIC3 KO mice).

The basal von Frey sensitivity of HT neurons in rats, determined with filaments ranging from 1 to 300 g, was also enhanced after LPC cutaneous injection (Figure 3F, From 0.62 ± 0.21 to 83.55 ± 6.24 spikes in control condition, and from 0.87 ± 0.40 to 131.93 ± 18.23 spikes after LPC16:0 injection), demonstrating a significant LPC-induced mechanical hypersensitivity from filaments ≥ 26 g. Similar results were also observed for rat WDR neurons from filaments ≥8 g (Supplementary Figure 5F, From 4.58 ± 0.80 to 83.73 ± 6.93 spikes in control condition, and from 4.20 ± 1.75 to 146.67 ± 21.15 spikes after LPC16:0 injection). Mechanical hypersensitivity in rats was prevented by the co-administration of the ASIC3 blocker APETx2 with LPC into the receptive fields of both HT (Figure 3F, From 0.76 ± 0.42 to 88.10 ± 14.18 spikes after LPC16:0 + APETx2 injection) and WDR neurons (Supplementary Figure 5F, From 11.77 ± 2.10 to 99.44 ± 14.72 spikes after LPC16:0 + APETx2 injection), fully consistent with a role of peripheral ASIC3 channels in cutaneous LPC effects.

### Lysophosphatidyl-Choline-Induced Hypersensitivity of Spinal High Threshold Neurons Is Not Restricted to Mechanical Stimuli

**FIGURE 4:**
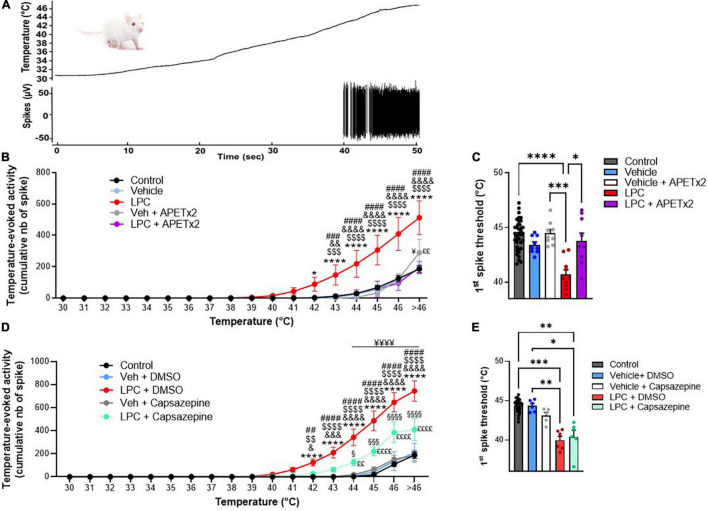
Effect of cutaneous LPC injection on heat sensitivity of spinal HT neuron. **(A)** Typical trace of a rat HT neuron response following heat ramp stimulation. Heat ramps from 30 to 46°C were applied onto neuron receptive field (top panel, black curve) while neuronal evoked activity was recorded (bottom panel). **(B)** Cumulative representation of the number of spikes evoked by heat ramps as a function of the temperature. Experiments were performed before (control, black dots, *n* = 37 neurons from 17 rats) and 20 min after cutaneous injection of vehicle (light blue dots, *n* = 9 neurons from 6 rats), LPC16:0 (red bar dots, *n* = 10 neurons from 6 rats), vehicle + APETx2 (gray dots, *n* = 9 neurons from 6 rats) or LPC16:0 + APETx2 (purple dots, *n* = 9 neurons from 7 rats; two-way ANOVA with *p* < 0.0001 for both treatment and temperature effects, followed by a Tukey’s multiple comparison test: *p < 0.05 and ****p < 0.0001 for LPC vs. control; $$$ *p* < 0.001 and $$$$ *p* < 0.0001 for LPC vs. vehicle; && *p* < 0.01 and &&&& *p* < 0.0001 for LPC vs. LPC + APETx2; ### *p* < 0.001 and #### *p* < 0.0001 for LPC vs. vehicle + APETx2; ¥ *p* < 0.05 for vehicle vs. vehicle + APETx2; ££ *p* < 0.01 for control vs. vehicle + APETx2). **(C)** Histogram of temperature thresholds that triggered the first spiking activity of HT neurons in response to heat (*n* = 37, 9, 10, 9, 9 and for control, vehicle, LPC, LPC + APETx2 and vehicle + APETx2, respectively, Kruskal-Wallis test with *p* < 0.0001, followed by a Dunn’s multiple comparison test: *p < 0.05, ***p < 0.001, and ****p < 0.0001). **(D)** Cumulative representation of the number of spikes evoked by heat ramps as a function of the temperature. Experiments were performed before (control, black dots, *n* = 23 neurons from 12 rats) and 20 min after cutaneous injection of vehicle + DMSO (light blue dots, *n* = 6 neurons from 4 rats), LPC16:0 + DMSO (red bar dots, *n* = 6 neurons from 5 rats), vehicle + capsazepine (gray dots, *n* = 5 neurons from 4 rats), or LPC16:0 + capsazepine (green dots, *n* = 6 neurons from 5 rats; two-way ANOVA with *p* < 0.0001 for both treatment and temperature effects, followed by a Tukey’s multiple comparison test: ****p < 0.0001 for LPC + DMSO vs. control; & *p* < 0.05, &&& *p* < 0.001, and &&&& *p* < 0.0001 for LPC + DMSO vs. LPC + capsazepine; $$ *p* < 0.01 and $$$$ *p* < 0.0001 for LPC + DMSO vs. vehicle + capsazepine; ## *p* < 0.01 and #### *p* < 0.0001 for LPC + DMSO vs. vehicle + DMSO; § *p* < 0.05, §§§ *p* < 0.001, and §§§§ *p* < 0.0001 for LPC + capsazepine vs. vehicle + capsazepine; ¥¥¥¥ *p* < 0.0001 for LPC + capsazepine vs. control; ££ *p* < 0.01 and ££££ *p* < 0.0001 for LPC + capsazepine vs. vehicle + DMSO). **(E)** Histogram of temperature thresholds that triggered the first spiking activity of HT neurons in response to heat (*n* = 23, 6, 6, 6, 5 and for control, vehicle + DMSO, LPC + DMSO, LPC + capsazepine and vehicle + capsazepine, respectively, Kruskal-Wallis test with *p* < 0.0001, followed by a Dunn’s multiple comparison test: *p > 0.05, **p < 0.01 and ***p < 0.001).

**FIGURE 5:**
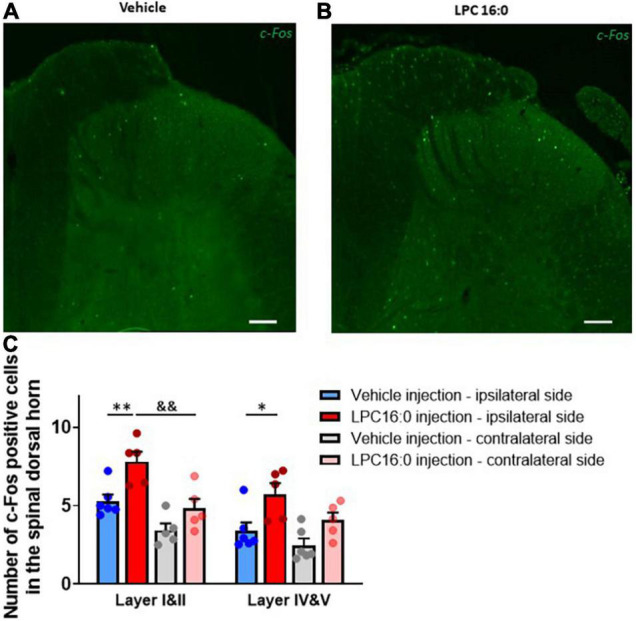
C-Fos immunohistochemistry in dorsal spinal cord. **(A,B)** Representative photomicrographs of c-Fos labeling following vehicle **(A)** and LPC16:0 **(B)** peripheral injection. Scale bar: 100 μm. **(C)** Quantification of the number of c-Fos positive cells in the spinal dorsal horn in I&II and IV&V spinal cord layers. Quantifications were done on both ipsilateral and contralateral spinal cord side after peripheral injections of LPC 16:0 (*n* = 5 for both) and vehicle (*n* = 6 and 5, respectively). Two-way ANOVA with *p* < 0.0001 for treatment effects, followed by a Tukey’s multiple comparison test: *p < 0.05; **p < 0.01 for LPC 16:0 injection vs. vehicle injection and &&*p* < 0.01 for ipsilateral vs. contralateral side.

Thermal sensitivity of spinal HT neurons was also tested to determine whether LPC induced-sensitization was dependent of the stimulus modality. Heat temperature ramps were applied onto rat HT neuron receptive fields, before and after LPC or vehicle injections (Figure 4A). As expected in control condition, the number of spike emitted by HT neurons increased as a function of temperature (from 0 spikes at 30°C to 186.08 ± 22.70 spikes at temperatures above 46°C; Figure 4B control). The discharge pattern was significantly enhanced following LPC16:0 cutaneous injection, especially for temperatures above 42°C (from 88.75 ± 43.82 spikes at 42°C to 513.05 ± 106.69 spikes at temperatures above 46°C; Figure 5B), compared to both control (from 2.22 ± 1.28 spikes at 42°C to 186.08 ± 22.67 spikes at temperatures above 46°C; Figure 5B) and vehicle injection (from 1.06 ± 1.06 spikes at 42°C to 187.61 ± 36.24 spikes at temperatures above 46°C; Figure 4B). Co-injection of APETx2 prevented LPC-induced thermal hypersensitivity, similarly to what has been observed for mechanical hypersensitivity. Thus, thermal-evoked activity of HT neurons was significantly reduced in the LPC16:0 + APETx2 condition (from 4.06 ± 3.63 spikes at 42°C to 196.44 ± 37.84 spikes at temperatures above 46°C; Figure 5B) compared to LPC16:0 alone (from 88.75 ± 43.82 spikes at 42°C to 513.05 ± 106.69 spikes at temperatures above 46°C; Figure 4B). Finally, a significant decrease of the temperature threshold triggering HT neuron’s spiking was also observed following LPC cutaneous injection (40.7 ± 0.4°C for LPC16:0 vs. 44.3 ± 0.2°C and 43.4 ± 0.3°C for control and vehicle, respectively; Figure 4C), which was also abolished by the co-injection of APETx2 (43.8 ± 0.7°C for LPC16:0 + APETx2; Figure 4C).

Finally, we assessed the potential role of TRPV1 channels in heat hypersensitivity of spinal HT neurons following LPC cutaneous injection. Capsazepine had no effect on basal heat sensitivity, but partially and significantly prevented LPC-induced thermal hypersensitivity of spinal HT neurons (Figure 4D). The lack of effect of capsazepine on the basal heat sensitivity of HT neurons does not exclude the involvement of TRPV1 that was previously demonstrated (Caterina et al., 2000), but rather illustrates the low efficacy of the drug on channel responses to heat (Savidge et al., 2001). Nevertheless, the use of capsazepine demonstrates an involvement of peripheral TRPV1 channels to LPC-induced heat hypersensitivity of spinal HT neurons (Figure 4D). Indeed, thermal evoked activity after LPC16:0 + capsazepine cutaneous injection (from 27.75 ± 12.28 spikes at 42°C to 409.08 ± 93.12 spikes at temperatures above 46°C) was significantly reduced compared to LPC16:0 alone (from 123.66 ± 29.73 spikes at 42°C to 745.00 ± 87.74 spikes at temperatures above 46°C), but was still significantly increased compared to control (from 0 spikes at 42°C to 188.33 ± 28.15 spikes at temperatures above 46°C). As observed previously (Figure 4C), LPC significantly decreased the temperature threshold triggering HT neuron’s spiking compared to control (Figure 4E, 44.3 ± 0.2°C vs. 40.0 ± 0.5°C for control and LPC16:0, respectively), but this effect was not reduced by capsazepine (Figure 4E, 40.4 ± 0.8°C for LPC16:0 + capsazepine).

### Lysophosphatidyl-Choline-Induced Hypersensitivity of Spinal High Threshold Neurons Displays Some, but Not All, Central Sensitization Features

To characterize the mechanism by which peripheral LPC affects spinal dorsal horn neuron activity, we assessed c-Fos expression in the lumbar spinal cord following cutaneous LPC injection (Figure 5). C-Fos expression was significantly increased in both ipsilateral layers I & II and layers IV & V of the spinal cord following LPC administration (Figure 5C), consistent with the increased neuronal activity observed for *in vivo* electrophysiological recordings. The number of c-Fos-positive cells was significantly higher after LPC cutaneous injection (Figures 5A,C, 7.81 ± 0.63 cells for layers I & II, 5.75 ± 0.70 cells for layers IV & V) compared to vehicle (Figures 5B,C, 5.29 ± 0.41 cells for layers I & II, 3.40 ± 0.54 cells for layers IV & V). C-Fos positive cell number after LPC peripheral injection was also significantly different between the ipsilateral (7.81 ± 0.63 cells) and contralateral (4.82 ± 0.61 cells) sides in layer I & II of the dorsal horn, suggesting that spinal neurons on the contralateral side were not activated by ipsilateral LPC injection.

**FIGURE 6:**
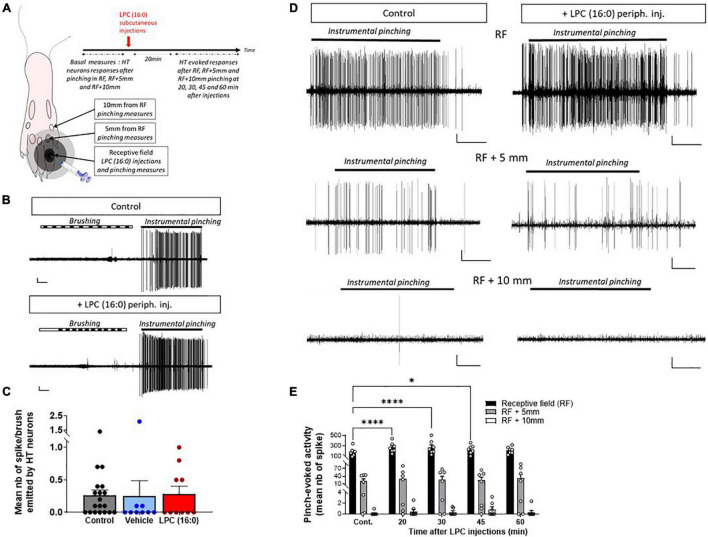
Effect of cutaneous LPC injection on the size of HT neuron receptive field. **(A)** Experimental protocol used to test the possible enlargement of rat spinal HT neuron receptive fields (RF). Different areas were initially determined: RF, RF + 5 mm and RF + 10 mm. Noxious pinches (instrumental pinching, 300 g) were then applied onto these different areas. **(B)** Typical traces of a HT neuron not responding to brush but to instrumental pinching (indicating by black lines) before (top panel) and after LPC16:0 peripheral injection (bottom panel). Scale bars: 50 μV–1 s. **(C)** Population data showing non-noxious responses of HT neuron before (gray bar, *n* = 19 neurons/19 rats) and after vehicle (blue bar, *n* = 9 neurons/9 rats) or LPC16:0 (red bar, *n* = 10 neurons/10 rats) peripheral injections. Kruskal and Wallis test with *p* = 0.4190. **(D)** Representative recordings of HT neuronal discharge following pinching of RF, RF + 5 mm, or RF + 10 mm areas before (left panels) and 20 min after LPC16:0 peripheral injection (right panels, scale bars: 10 μV–1 s). **(E)** HT neuronal responses following RF (black bar), RF + 5 mm (gray bar) and RF + 10 mm (white bar) noxious stimulations, before and after LPC16:0 cutaneous injection. LPC only enhanced HT neurons evoked response when pinches were applied within the RF and not at RF + 5 mm or RF + 10 mm (*n* = 7 neurons from 7 rats, two-way ANOVA with *p* = 0.0043 and *p* < 0.0001 for time after LPC and RF area effects, followed by a Dunnet’s multiple comparison test: *p < 0.05 and ****p < 0.0001 as compared to control).

Additional experiments have been made to determine whether neuronal activation in the spinal cord following peripheral LPC cutaneous injection could induce central sensitization features. Among the features particular to central sensitization, we already demonstrated hyperexcitability of spinal HT and WDR neurons (Figures 1–4). We then studied two other features that could be possibly affected by LPC (Figure 6A): the conversion of nociceptive-specific neurons to WDR neuronal type (Figures 6B,C), as well as the enlargement of the receptive field of spinal neurons (Figures 6D,E) that are typically associated with central sensitization (Latremoliere and Woolf, 2009).

Nociceptive-specific spinal neurons, i.e., HT neurons, only respond to noxious stimuli in control condition. If central sensitization is induced in spinal HT neurons following LPC cutaneous injection, they should be converted to WDR neurons and also respond to non-noxious stimulation such as brushing. The number of spikes emitted by HT neurons in response to non-noxious stimulations after LPC cutaneous injection (Figures 6B,C) was not significantly different from control and vehicle (Figure 6C), indicating that HT neurons were not converted to WDR neurons in our conditions.

Receptive fields were defined as the areas inside which noxious pinching induced strong responses of HT neurons before LPC injection (Figures 6A,D, upper-left trace). Noxious stimulations were then applied 5 mm and 10 mm outside the receptive field (Figure 6A). In control condition, HT neuronal responses to pinch were weak 5 mm and absent 10 mm away from the receptive field (Figure 6D, Middle and bottom left traces). Following LPC cutaneous injection, HT neuronal responses were significantly increased following stimulation within their receptive field (Figure 6D, Upper-right trace and Figure 6E), as observed previously (Figures 2, 4). However, no significant change was observed in our conditions when stimulations were applied 5 mm or 10 mm away from the receptive field (Figure 6D, Middle- and bottom-right traces, and Figure 6E). However, the dose of LPC used in these experiments has been identified based on dose-dependency of the pinch-evoked activity of HT neurons (Supplementary Figure 1B), and it remains possible that a transition of HT to WDR and/or an increase of receptive field could occur at higher (or lower) doses of LPC.

## Discussion

The production of LPC *via* the activation of phospholipase A2 (PLA2), oxidative stress, or as an intermediate product of phosphatidylcholine metabolism occurs in many tissues, including the nervous system. Interestingly, elevated levels of LPC have been detected in the synovial fluids and plasma of patients suffering from rheumatic diseases and fibromyalgia (Hung et al., 2020; Jacquot et al., 2022), where they were correlated with patient pain symptoms, supporting a peripheral role of this endogenous phospholipid in pain in humans. Moreover, local injection of LPC induces acute and chronic pain in rodents through its effects on pain-related ion channels, including some TRPs and ASIC3 (Gentry et al., 2010; Marra et al., 2016; Hung et al., 2020; Rimola et al., 2020; Sadler et al., 2021; Jacquot et al., 2022). The aim of this work was to (i) study the pain message generated by peripheral LPC when injected in the skin, (ii) determine the contribution of peripheral ASIC3 to the generation of this message and, (iii) investigate how this message is processed by spinal dorsal horn neurons.

Our data show that peripheral cutaneous LPC injection exclusively affects the nociceptive pathway by inducing an ASIC3-dependent sensitization of peripheral nociceptive fibers, ultimately leading to increased spontaneous and evoked-activities of spinal HT and WDR neurons. The activity of spinal LT neurons remained unaffected by cutaneous injection of LPC, consistent with a lack of effect of this lysolipid on non-nociceptive fibers. The sensitizing effect of LPC on HT and WDR neurons occurs following a single cutaneous injection and lasts approximately 45 min. It is also modality-independent since neuronal responses to noxious heat and mechanical stimulations are both potentiated. This is consistent with the recent description of an acute mechanical hypersensitivity after cutaneous injection of LPC in mice (Rimola et al., 2020). Basal mechanical and thermal sensitivities are not affected by either pharmacological or genetic inhibition of ASIC3, in agreement with previous studies using ASIC3 KO mice to test behavioral responses following noxious thermal or mechanical stimulations (Price et al., 2001; Chen et al., 2002; Borzan et al., 2010). However, the mechanical and thermal hypersensitivity of spinal HT neurons induced by cutaneous injection of LPC is clearly dependent on peripheral ASIC3 activation. The persistent depolarizing ASIC3 current generated by LPC is likely to participate in this ASIC3-dependent sensitization of nociceptive fibers, as demonstrated in primary cultures of dorsal root ganglia neurons (Deval et al., 2003; Jacquot et al., 2022). Interestingly, LPC does not seem to produce the same effect on non-nociceptive fibers where ASIC3 is also largely expressed (Molliver et al., 2005; Lin et al., 2016), suggesting some cell-specific roles of this channel. The augmented peripheral nociceptive inputs is likely to drive the increase of spinal activities from HT and WDR neurons.

Lysophosphatidyl-choline displays a good specificity for ASIC3 compared to other pain-related ASICs also expressed in peripheral sensory neurons such as ASIC1a and ASIC1b, as shown here and in Marra et al. (2016). LPC not only modulates ASIC3 (Marra et al., 2016), but also affects other pain-related channels, including TREK1 and TRAAK (Maingret et al., 2000), TRPM8 (Andersson et al., 2007; Gentry et al., 2010), TRPC5 (Flemming et al., 2006; Sadler et al., 2021), and TRPV1 (Rimola et al., 2020). Our data demonstrated that at least TRPV1 and TRPM8 channels did not significantly contribute to the effect of LPC on HT neuron mechanical hyperexcitability. However, TRPV1 was involved in heat thermal hypersensitivity, as expected for this heat-sensitive channel. Indeed, pharmacological experiments with capsazepine and APETx2 are consistent with a direct role of TRPV1 in thermal transduction and a more pivotal role of ASIC3 in setting the overall neuronal excitability. This does not, however, preclude the participation of other LPC-modulated channels to the cutaneous effects of this lipid in physiological or pathophysiological conditions.

An increase spinal activity can result from both peripheral and central sensitization processes. Spinal dorsal horn neurons subject to central sensitization exhibit typical features, such as increased spontaneous activity, lower activation threshold to peripheral stimuli, increase response to suprathreshold stimulations, and an enlargement of the neuronal receptive field (Latremoliere and Woolf, 2009). We demonstrate here that spinal HT and WDR neurons exhibited enhanced spontaneous activities following LPC cutaneous injections as well as reduced temperature threshold triggering spikes in HT neurons. Moreover, the facilitation process of windup was potentiated by LPC. On the other hand, experiments using von Frey stimuli did not reveal any significant responses of HT and WDR neurons to subthreshold stimulations, at least for the filaments used that were at or below rat and mouse thresholds. It seems therefore that a single LPC subcutaneous injection would elicit rather hyperalgesia than allodynia. Finally, the increased response of spinal neurons to suprathreshold stimuli is rather short (45 min) and, importantly, no enlargement of spinal neuron receptive fields, nor conversion of HT to WDR neuron or bilateral activation of spinal dorsal horn neurons have been observed at the dose of LPC used. However, we cannot completely exclude such phenomena at different doses. Thus, spinal neuron activity displayed some, but not all, the features of central sensitization, rather suggesting short-term than long-term central sensitization following subcutaneous LPC. This effect is driven by peripheral ASIC3 channels activation, which increase nociceptive inputs, leading to the enhancement of both spontaneous firing and evoked responses of spinal neurons to noxious stimuli.

It is interesting to compare the effects of LPC subcutaneous injection with those recently reported for intra-articular or intra-muscular administrations of LPC (Hung et al., 2020; Jacquot et al., 2022). If the single subcutaneous injection of LPC described here only induces short-term hypersensitivity of the nociceptive pathway, in agreement with other studies (Gentry et al., 2010; Rimola et al., 2020), a single knee injection of LPC generates a secondary mechanical allodynia lasting for several days (Jacquot et al., 2022). Most importantly, two consecutive injections of LPC within muscles or joints induce chronic pain states associated to a sensitization of spinal HT neurons (Hung et al., 2020; Jacquot et al., 2022), in agreement with the high level of LPC detected in patients with established joint or muscle painful diseases (Marra et al., 2016; Hung et al., 2020; Jacquot et al., 2022). Such a difference in LPC effects between skin and joint/muscle may be related to different ASIC3 levels in the peripheral fibers innervating these tissues (Molliver et al., 2005), and/or to different processing of the pain information associated with superficial and deep tissues (Sluka, 2002; Prato et al., 2017).

Our study demonstrates how a single cutaneous injection of LPC can generate a short-term peripheral sensitization of nociceptive fibers. The underlying mechanism mainly involves pain-related ASIC3, but also TRPV1 channels, which can be both activated by this lipid. The nociceptive input induced by a single LPC cutaneous injection did not appear to be sufficient to drive long-term spinal synaptic plasticity, contrary to injections in muscle and joint (Hung et al., 2020; Jacquot et al., 2022). If LPC effects on nociceptive pathways clearly depend on peripheral ASIC3 channels, their consequences on pain may be tissue-dependent.

## Data Availability Statement

The raw data supporting the conclusions of this article will be made available by the authors, without undue reservation.

## Ethics Statement

This study was reviewed and approved by CIEPAL-Azur and the French Ministry of Research, approval number 02595.02.

## Author Contributions

LP: conception and design, acquisition, and analysis and interpretation of *in vivo* data. KD: acquisition and analysis and interpretation of *in vitro* data. EL: interpretation of data. JB and FM: perform c-Fos immunohistochemistry. ED: conception and design and analysis and interpretation of data. All the authors have contributed to drafting the manuscript and have given approval to its final version.

## Conflict of Interest

The authors declare that the research was conducted in the absence of any commercial or financial relationships that could be construed as a potential conflict of interest.

## Publisher’s Note

All claims expressed in this article are solely those of the authors and do not necessarily represent those of their affiliated organizations, or those of the publisher, the editors and the reviewers. Any product that may be evaluated in this article, or claim that may be made by its manufacturer, is not guaranteed or endorsed by the publisher.
