# Supplementary material for: Single Subcutaneous Injection of Lysophosphatidyl-Choline Evokes ASIC3-Dependent Increases of Spinal Dorsal Horn Neuron Activity
Source: Front Mol Neurosci. 2022 Jun 14;15:880651. doi: 10.3389/fnmol.2022.880651 (PMC9239072; doi:10.3389/fnmol.2022.880651)
Supplement: Supplementary file 1 [file Data_Sheet_1.pdf]

Supplementary Material

1 Supplementary Figures and Tables

1.1 Supplementary Figures

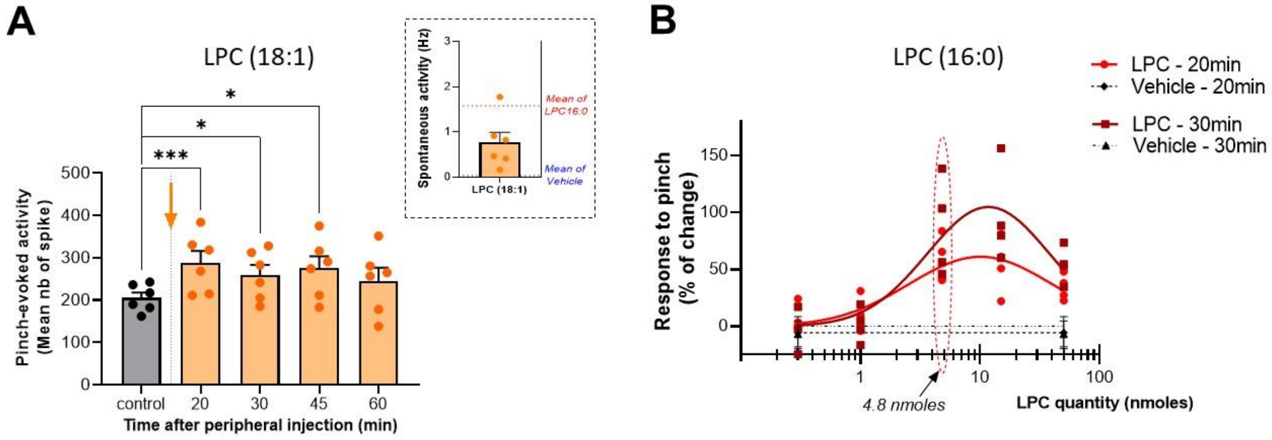

Supplementary Figure 1. Effects of cutaneous injection of LPC16:0 or LPC18:1 on spontaneous and pinch-evoked activity of spinal HT neurons. (A) Pinch-evoked responses of spinal HT neurons (instrumental pinching, 300 g) before (black bar and point) and after LPC18:1 (orange bar and point) subcutaneous injection in their receptive fields (n = 6 neurons from 3 rats, Friedman test with $p = 0.0009$ followed by a Dunn's multiple comparison test: * $p < 0.05$ and *** $p < 0.001$). The potentiation by cutaneous injection of LPC18:1 was still significant 45 min after injection. Inset: global spontaneous discharge of HT neurons after LPC18:1 (orange bar and points). For reminder, mean global discharge after LPC 16:0 (red dotted line) and vehicle (blue dotted line) were reported on the graph. **(B)** LPC16:0 dose-response curves measured 20 min (red) and 30 min (brown) after peripheral injections. Dose-response curves for LPC16:0 were obtained using five different doses in rats (*i.e.*, 0.3, 1, 4.8, 15 and 50 nmoles diluted in NaCl 0.9%). Response to pinch was expressed as the percent change of the number of spikes emitted after LPC (red and brown) or vehicle (black) peripheral injection versus the number of spikes emitted before injection.

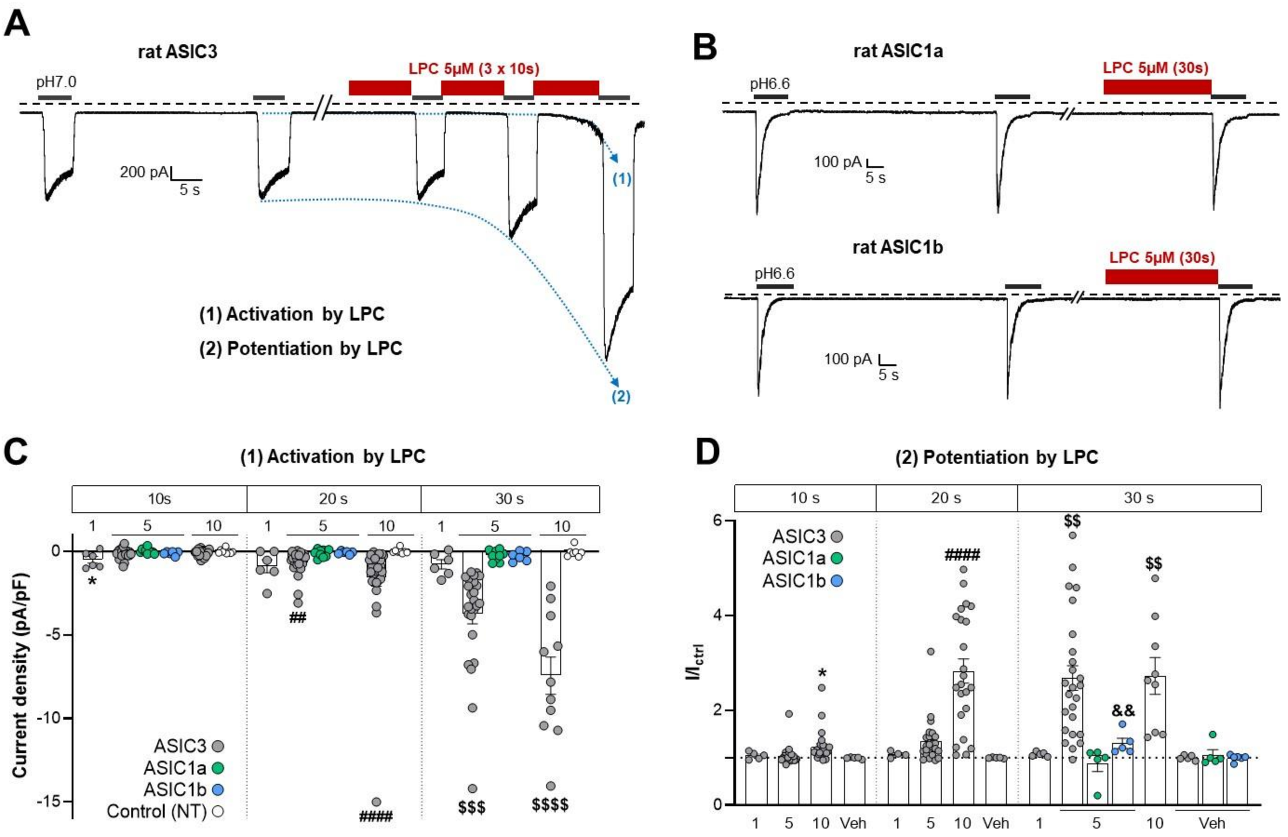

Supplementary Figure 2. *In vitro* effects of LPC on ASIC1 and ASIC3 channels. (A) Representative current trace recorded at -80 mV from HEK293 cells transfected with rat ASIC3. Currents were elicited by repetitive extracellular pH drops (from pH 7.4 to pH 7.0 every 10 s), and LPC16:0 (5 µM) was applied extracellularly for 30 s, as indicated above each traces (red bar). The protocol allowed studying the effects of LPC on both basal activation of ASIC3 (1) and potentiation of its acid-evoked currents (2). **(B)** Representative current traces recorded at -80mV from HEK293 cells transfected with rat ASIC1a (upper trace) or rat ASIC1b (lower trace) cDNAs. Currents were elicited by repetitive extracellular pH drops (from pH 7.4 to pH 6.6 every 60 s) and LPC16:0 (5µM) was applied extracellularly for 30 s, as indicated above each traces. **(C)** Bar graphs showing the mean current densities (pA/pF) activated by different concentrations of LPC16:0 (1 µM, 5 µM and 10 µM) after 10, 20 and 30 s application on ASIC1a, ASIC1b and ASIC3 transfected cells (n = 6-29; *, p < 0.05 compared to control non-transfected cells (NT) at 10 s; ##, p < 0.01 and ####, p < 0.0001 as compared to control NT at 20 s; $$$, p < 0.001 and $$$$, p < 0.0001 as compared to control NT at 30 s, Kruskal-Wallis tests followed by Dunn's multiple comparison tests). **(D)** Bar graphs showing the potentiation of pH-evoked ASIC currents (pH 7.0 for ASIC3 and pH6.6 for ASIC1a and ASIC1b) by different concentrations of LPC16:0 (1 µM, 5 µM and 10 µM) after 10, 20 and 30 s application on ASIC1a, ASIC1b and ASIC3 transfected cells. The pH-evoked current amplitudes are normalized (I/Ictrl) to the current measured before extracellular application of LPC16:0 (n = 4-25; *, p < 0.05 compared to control vehicle at 10 s; ###, p < 0.001 compared to control vehicle at 20 s; $$, p < 0.01 compared to control vehicle for ASIC3 at 30 s; &&, p < 0.01 compared to control vehicle for ASIC1b at 30 s, Mann-Whitney test).

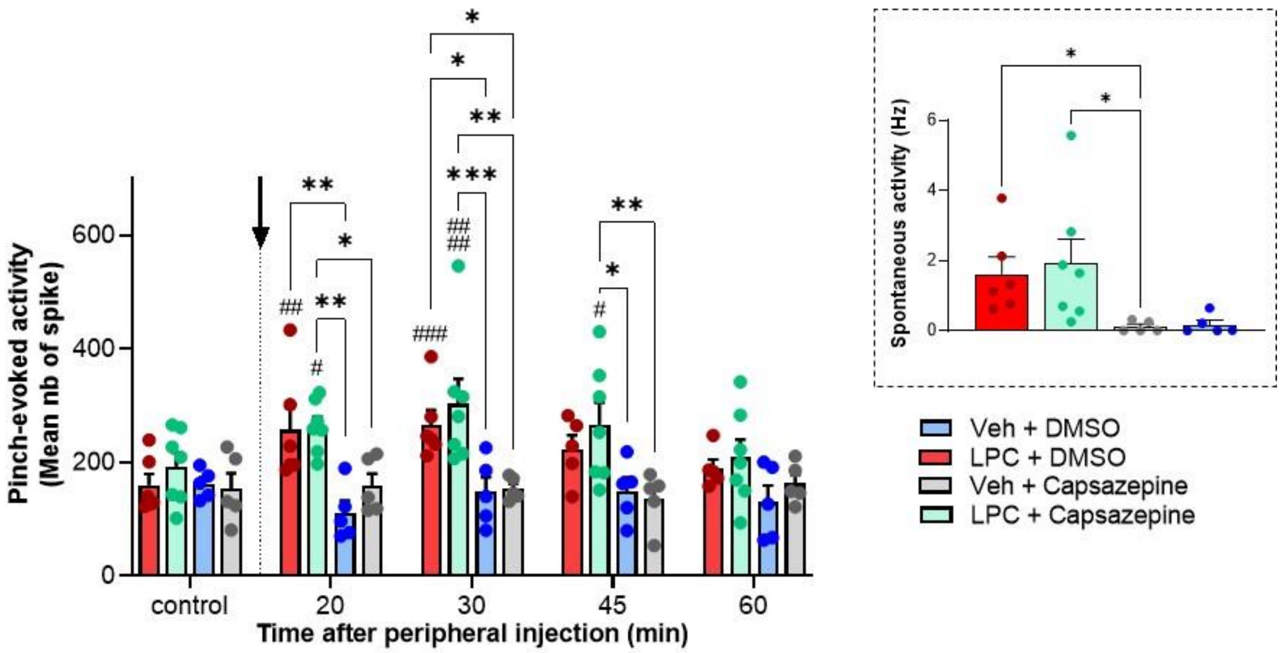

Supplementary Figure 3. Effect of capsazepine on spontaneous and pinch-evoked activity of spinal HT neurons following subcutaneous injection of LPC. Pinch-evoked activity of rat HT neurons measured before and after subcutaneous injection of LPC16:0 and/or capsazepine, or their vehicles (n = 6, 7, 5, 5 neurons for LPC16:0 + DMSO, LPC16:0 + capsazepine, vehicle + capsazepine and vehicle + DMSO respectively; two-way ANOVA (mixed effect model) with $p = 0.0071$ and $p = 0.0025$ for treatment and time after injection, respectively; Dunnet's multiple comparison post hoc test: #, $p < 0.05$, ##, $p < 0.01$, ###, $p < 0.001$ and ###, $p < 0.0001$ compared to respective control; Tukey's multiple comparison post hoc test: *, $p < 0.05$, **, $p < 0.01$ and ***, $p < 0.001$). The potentiation induced by LPC16:0 + capsazepine was still significant 45 min after its subcutaneous injection, and was not different from that of LPC16:0 + DMSO. Note that capsazepine had no effect by itself (vehicle + capsazepine) on the pinch-evoked activity of HT neurons. Pinch-evoked activity was potentiated by +36.4%, +57.3% and + 38.5% after LPC16:0 + capsazepine compared to control at 20, 30 and 45 min, respectively, and by +60.7%, +66.5% and +39.3% after LPC16:0 + DMSO compared to control at 20, 30 and 45 min. Inset: Spontaneous discharge of HT neurons after vehicle + DMSO (blue bar and points), LPC16:0 + DMSO (red bar and points), vehicle + capsazepine (grey bar and points), and LPC16:0 + capsazepine (green bar and points) injection (n = 6, 7, 5, 5 neurons for LPC16:0 + DMSO, LPC16:0 + capsazepine, vehicle + capsazepine and vehicle + DMSO respectively; Kruskal-Wallis test with $p = 0.0024$ followed by a Dunn's multiple comparison test: *, $p < 0.05$).

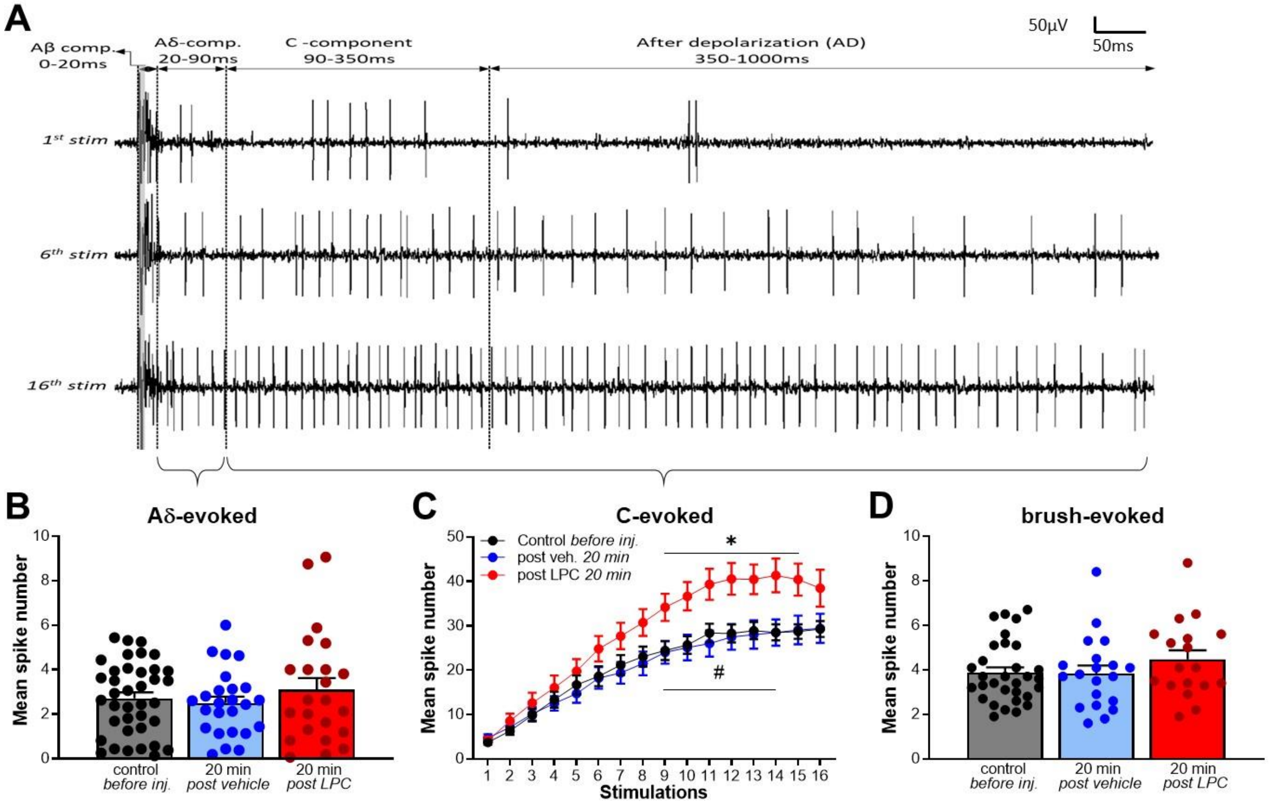

Supplementary Figure 4. Effect of LPC subcutaneous injection on WDR neuron evoked activity. (A) Typical recordings obtained from a rat WDR neuron after the 1st (top trace), the 6th (middle trace) and the 16th (bottom trace) electrical stimulation of its receptive field. Windup was induced by 16 supra-threshold electrical stimulations at 1Hz (see Methods). Vertical dotted lines delimit the four periods used to distinguish the WDR spiking evoked by Aβ (0-20 ms), Aδ (20-90 ms) and C fibers (90-350 ms), and with the spikes emitted during the 350-1,000 ms interval attributed to the after depolarization (AD) period. If Aβ-evoked activity is difficult to distinguish because emitted spikes are mixed with stimulation artifacts, activities related to Aδ- and C-fibers can be easily measured. **(B-D)** Pooled data regardless of the protocol used (1 and 2, see Fig. 1A). Part of these data was already presented in Fig. 2. **(B)** Twenty minutes after its cutaneous injection, LPC16:0 had no significant effect on the WDR spiking activities evoked by Aδ inputs (n = 38, 25 and 23, for control, vehicle and LPC16:0, respectively; Kruskal-Wallis test with $p = 0.8026$). **(C)** Windup curves representing the number of emitted spikes during the C-fiber + AD periods as a function of the electrical stimulation number, before and 20 min after vehicle or LPC16:0 cutaneous injection (n = 38, 25 and 23 for control, vehicle and LPC, respectively; two-way ANOVA test with $p = 0.0128$ and $p < 0.0001$ for treatment and stimulation number, respectively; Tukey's post hoc test: *, $p < 0.05$ and #, $p < 0.05$ for control *vs.* LPC16:0 and vehicle *vs.* LPC16:0, respectively). **(D)** Mean number of spikes evoked by non-noxious brushings (n = 31, 20 and 18, for control, vehicle and LPC16:0, respectively; Kruskal-Wallis test with $p = 0.4528$).

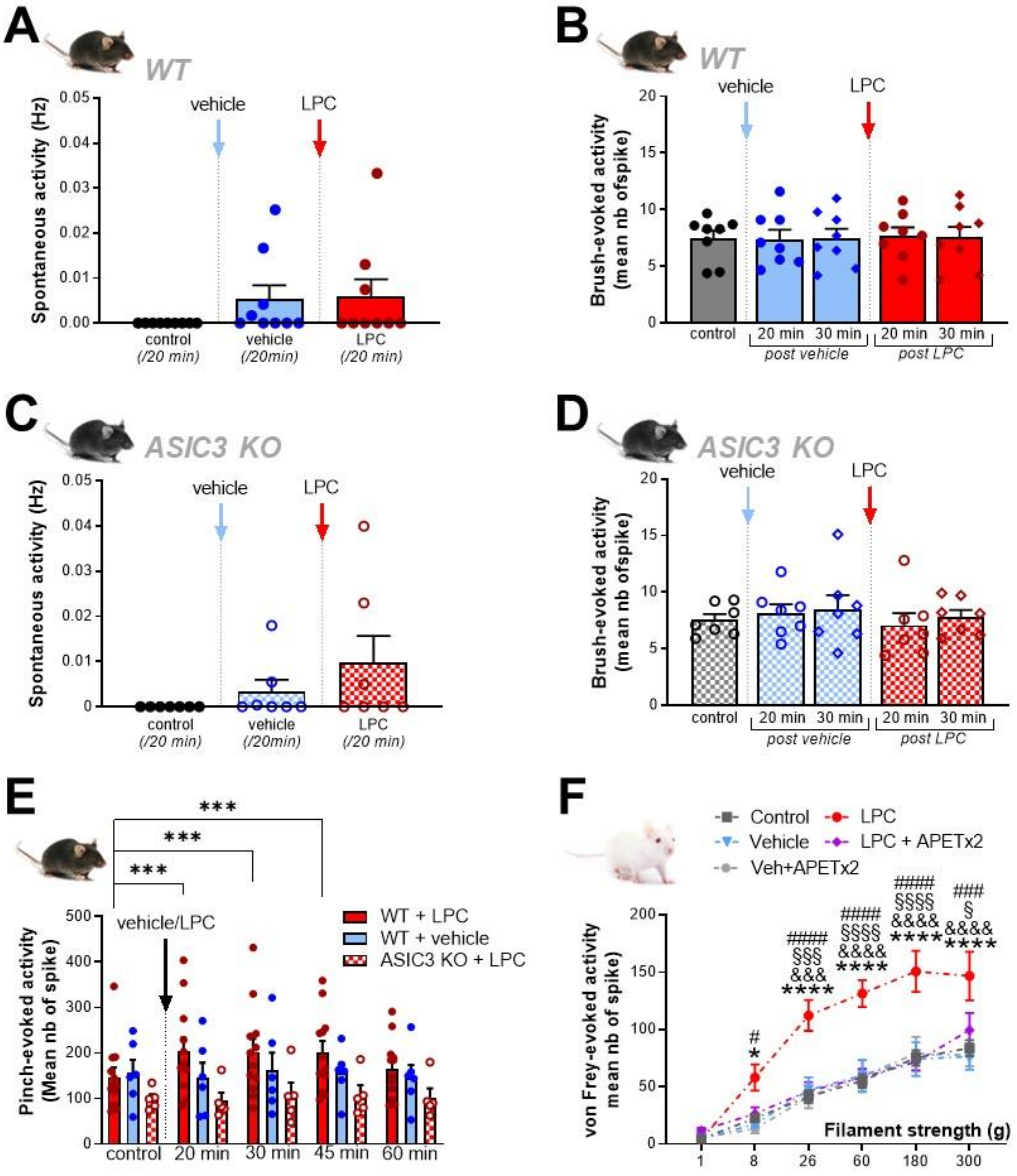

Supplementary Figure 5. Effect of LPC subcutaneous injection on mechanical sensitivity of spinal LT, HT and WDR neurons. (A) Global spontaneous discharge of LT neurons (n = 9 neurons from 5 WT mice) before and after vehicle (blue bar and points) and LPC16:0 (red bar and points) subcutaneous injections in the hindpaw receptive fields (no significant difference, Friedman test with p = 0.1728). **(B)** Evoked response of LT neurons to non-nociceptive stimulation (brushing) before and after vehicle (blue bar and points) and LPC16:0 (red bar and points) subcutaneous injection. Effects

were assessed at 20 min (circle point) and 30 min (diamond point) after injection (n = 8 neurons from 4 mice, Friedman test with p = 0.8687). **(C-D)** Spontaneous activity (C) and brush-evoked responses (D) of ASIC3 KO LT neurons (n = 7 neurons from 4 mice, no significant difference with p = 0.1944 (C) and p = 0.1751 (D), Friedman tests). **(E)** Duration of LPC16:0 effect on brush-evoked responses of mouse HT neurons. Part of the data were pooled from different experiments already presented in Fig. 2, regardless of the protocol used (n=12 neurons from 10 mice, 5 neurons from 4 mice, and 6 neurons from 6 mice for WT + LPC16:0, ASIC3 KO + LPC16:0 and WT + vehicle, respectively; two-way ANOVA test with p = 0.0976 and p = 0.1197 for time and LPC treatment effects, respectively, followed by a Dunnet's multiple comparison test: ***, p < 0.001 as compared to control). **(F)** Mechanical sensitivity of rat WDR neurons to von Frey filaments before (control, n = 33 neurons from 24 rats) and after injection of vehicle (n = 8 neurons from 5 rats), LPC16:0 (n = 8 neurons from 7 rats), LPC16:0 + APETx2 (n = 9 neurons from 6 rats) and vehicle + APETx2 (n = 8 neurons from 6 rats; two-way ANOVA with p < 0.0001 for both treatment and von Frey filament effects, followed by Tukey's multiple comparison test: *, p < 0.05 and ****, p < 0.0001 for LPC *vs.* control; &&&, p < 0.001 and &&&&, p < 0.0001 for LPC *vs.* vehicle; §, p < 0.05, §§§, p < 0.001 and §§§§, p < 0.0001 for LPC *vs.* LPC + APETx2; #, p < 0.05, ###, p < 0.001 and ####, p < 0.0001 for LPC *vs.* vehicle + APETx2).
